# Season of Birth and Exceptional Longevity: Comparative Study of American Centenarians, Their Siblings, and Spouses

**DOI:** 10.4061/2011/104616

**Published:** 2011-11-30

**Authors:** Leonid A. Gavrilov, Natalia S. Gavrilova

**Affiliations:** Center on Economics and Demography of Aging, NORC at the University of Chicago, 1155 East 60th Street, Chicago, IL 60637, USA

## Abstract

This study explores the effects of month of birth (a proxy for early-life environmental influences) on the chances of survival to age 100. Months of birth for 1,574 validated centenarians born in the United States in 1880–1895 were compared to the same information obtained for centenarians' 10,885 shorter-lived siblings and 1,083 spouses. Comparison was conducted using a within-family analysis by the method of conditional logistic regression, which allows researchers to control for unobserved shared childhood or adulthood environment and common genetic background. It was found that months of birth have significant long-lasting effect on survival to age 100: siblings born in September–November have higher odds to become centenarians compared to siblings born in March. A similar month-of-birth pattern was found for centenarian spouses. These results support the idea of early-life programming of human aging and longevity.

## 1. Introduction

Studies of centenarians (persons living to age 100 and over) are useful in identifying factors leading to long life and avoidance of fatal diseases. These studies may be a sensitive way to find genetic, familial, environmental, and life-course factors associated with lower mortality and better survival [1, 2]. Several theoretical concepts suggest that early-life events and conditions may have a significant long-lasting effect on survival to advanced ages. These concepts include (but are not limited to) the idea of fetal origin of adult diseases also known as the Barker hypothesis [3, 4] and the related idea of early-life programming of aging and longevity; the theory of technophysio evolution [5], the reliability theory of aging, and the high initial damage load (HIDL) hypothesis in particular [6, 7]. These ideas are supported by the studies suggesting significant effects of early-life conditions on late-life mortality [3, 8–10]. Finch and Crimmins [11] suggested that historical decline in chronic inflammation (due to decreasing exposure to early-life infections) has led to a decrease in morbidity and mortality from chronic conditions at old age. They showed that both childhood mortality and cardiovascular diseases of old age may share common infectious and inflammatory causes rooted in the external environment [12].

Month of birth often is used by epidemiologists as a proxy characteristic for environmental effects acting during in-utero and early infancy development. These early effects include temperature and sun exposure during in-utero and early postnatal period, nutritional status during early development, exposure to infectious agents, and other factors [3, 13, 14]. Previous studies demonstrated that life expectancy may be influenced by person's month of birth [15–18]. However, studies of month-of-birth effects on longevity face significant difficulties in finding appropriate data on differential mortality by season of birth. Longitudinal data with information about season of birth are the optimal data for study of month-of-birth effects on longevity [19]. Such longitudinal data were available for population of Denmark and showed that the remaining life expectancy at age 50 was higher for persons born in October-November compared to persons born in April–June [15]. In other studies, the effects of month of birth on late-life mortality were estimated indirectly using information on mean age at death from cross-sectional collection of death certificates [19–22].

Little information is available on the month of birth association with exceptional longevity. To our knowledge, there is only one study that examines the effects of month of birth on longevity [23]. In this study, month-of-birth distribution of 925 age-validated German semi-supercentenarians (persons aged 105+ years) was compared to seasonal distribution of births in the German Empire at the time of semi-supercentenarians' birth (1880–1900). It was found that more semi-supercentenarians than expected were born in December while the proportion of semi-supercentenarians born in June was low. This study suggests that the December-born have a significantly higher risk of surviving up to age 105+ compared to the June-born [23] although it cannot be indicated unequivocally if month-of-birth pattern among semi-supercentenarians is due to seasonality of infant mortality or later-life month-of-birth effects. Additional problems in the studies of month-of-birth effects on longevity arise from possible confounding due to between-family variation in childhood socioeconomic conditions [24–26] and parental genetic background [27]. One possible solution to these challenges is to compare associations within sibships taking into account that socioeconomic and genetic background is similar for siblings from the same family [14, 28].

In this study, we analyze the effects of month of birth on survival to age 100 years using a large set of centenarians born in the United Sates in 1880–1895 and their shorter-lived siblings and spouses. Siblings share early childhood conditions including parental socioeconomic status, genetic background, and geographical location while spouses share common adulthood environment. It was shown that longevity has a significant familial component [29–32] suggesting the need to control for this important factor. Comparing month-of-birth characteristics of adult siblings or spouses with that of centenarians provides an opportunity for obtaining net effects of month-of-birth on survival and control for unobserved confounding factors.

## 2. Methods

### 2.1. Data Collection

This study compares centenarians to their shorter-lived siblings (who share common childhood conditions and genetic background) and spouses (who share common adulthood environment) using a large set of computerized family histories. Family histories (genealogies) proved to be a useful source of information for studies in historical demography [33] and biodemography [34, 35]. In this study, data were collected through a search of over 400,000 online family histories available at Rootsweb (http://wc.rootsweb.ancestry.com), which is one of the largest publicly available repositories of online genealogies. Search for centenarians in the Rootsweb database was conducted with assistance of the web-automation technique [36], which allows researchers to run automated queries (using program scripts in PHP language) and search online databases for individuals with desired properties (persons who lived 100+ years in our case). Applying this technique helps researchers to save time and effort on routine data collection from online resources. Application of the web-automation technique to the Rootsweb publicly available online resource identified over 40,000 records of centenarians born in 1880–1895 with known names of their parents. However, in many cases, one and the same centenarian appeared in two or more genealogies. After removing these duplicates, we obtained 23,127 records for centenarians born in 1880–1895 with detailed information on their birth and death dates as well as birth and death dates of their parents. According to the past experience with computerized genealogies [34], availability of detailed information on vital events ensures a good quality of collected genealogies. However, a significant proportion of records for siblings in the obtained genealogies did not contain information about death dates that we needed for the within-family analysis of human longevity. So the next step was to indentify the most informative families with complete information on birth and death dates for siblings. As a result of this identification procedure, we found 2,834 families where information on birth and death dates was known for more than 80 percent of siblings in a family. This procedure resulted in a set of families having higher-than-average sibship size and hence providing more control records (siblings) for the matched case-control study. During this data refining procedure, the proportion of male centenarians in genealogies dropped from 28.2% to 23.2% (see Table 1) and became close to the proportions reported in the USA censuses (19.3–24.0%) [37], which indicates an improvement in quality for the selected genealogies.

### 2.2. Data Verification

Previous studies demonstrated that age misreporting and age exaggeration in particular are more common among long-lived individuals [38, 39]. Therefore, the primary focus of data cleaning in this study was on the age verification for long-lived individuals. We followed the approach of age verification and data linkage [38, 40], which we applied previously on another dataset of centenarians [41]. This approach involves data consistency checks, death date verification through the linkage to the Social Security Administration Death Master File (DMF) and birth date verification through the linkage to early USA censuses. DMF is a publicly available data resource (available at the Rootsweb.com website), which covers deaths that occurred in the period 1937–2010 and captures about 95% of deaths recorded by the National Death Index [42]. More details about the procedure of centenarian age validation were published elsewhere [41]. Validation of centenarian death and birth dates produced 1,574 centenarians. Information on siblings and spouses of validated centenarians was collected using the web-automation technique described earlier. Table 1 shows the steps of data collection and cleaning for this study. Note that the proportion of males among validated centenarians found in genealogies (23%) is close to the official reports (19–24%) for centenarians in the United States based on the census data [37].

We used only those records of centenarians whose age was successfully confirmed through the DMF (with matched birth and death years). We added only few cases where death year was different from that found in the DMF (however, in these cases, the individual still had a centenarian status). Our previous work with centenarian data cleaning showed that incorrect death dates was the main source of errors in genealogical records of centenarians [41]. At the same time, birth dates were correctly reported in practically all records that had correct death dates and good consistency of birth and death dates for parents and siblings. Therefore, in this study we conducted a birth date verification procedure for a portion of approximately 15% of records. In all cases, birth years of centenarians agreed well with information reported in 1880, 1900, or 1910 censuses (as well as information about birth years of siblings). In addition to that, partial verification of centenarian birth dates was already accomplished through the linkage to DMF.

As a result of data quality checks, we found 1,574 records of centenarians born in 1880–1895 with verified birth and death dates. Given the fact that longevity is often clustered in families, we found other centenarians in the studied families (born outside the 1880–1995 time window) so that the total number of centenarians increased to 1,945 persons. Distribution of centenarians according to their lifespan is presented in Table 2. Note that the majority of centenarians lived less than 103 years and there are no claims of extraordinary high longevity (above 112 years) in the sample.

### 2.3. Life Span Data Reconstruction for Siblings and Spouses

Birth dates were reconstructed for all centenarian siblings using information available in computerized genealogies and early censuses. The procedure of death date verification using DMF is not feasible for validating death dates of shorter-lived siblings or spouses (used as controls) because data completeness of DMF is not very high for deaths occurred before the 1970s [43]. State death indexes, cemetery records, and obituaries cover longer periods of time. Taking into account that exact ages of death for controls (siblings) are not particularly important for comparison (it is sufficient to assume that they lived less than 100 years), we relied on death date information recorded in family histories for siblings and spouses not found in external sources. This approach was used previously in the Utah Population Database study for individuals died before 1932 [30]. Death dates were reconstructed for 99.99% of siblings using the social security death master file, state death indexes, and online genealogies (only 124 out of 13,654 cases were left unresolved).

### 2.4. Study Population

Data for 10,885 siblings of 1,574 centenarians were used in this study. As a result, each case (centenarian) had about 7 control siblings on average. The sibship size (eight siblings on average) in the studied centenarian families is higher than the average number of children in American families reported by the 1900 USA Census: 5.12 ± 0.01; data obtained from the 5% sample of the US 1900 Census from the integrated public use microdata series (IPUMS) [44]. Larger sibship size in the centenarian families compared to the general population can be explained by the fact that genealogies are more likely to be compiled for larger families and that longer-lived individuals in the United States were born more often in rural areas with higher fertility [41, 45]. This difference in sibship size with the general population is not critical for the within-family design of this study when appropriate control group (shorter-lived siblings raised in the same family or spouses) is selected.  Table 3 presents characteristics of the final sample used in this study. 171 siblings and 4 centenarians had unknown month of birth, so their records were excluded from the statistical analyses. As expected, spouses have higher age at death than siblings whose age at death was not conditioned on survival to ages eligible for marriage. Centenarians and siblings were born in about the same year on average. Spouses of male centenarians were approximately 5 years younger and spouses of female centenarians were about 3 years older on average than their long-lived mates (see Table 3). 5% sample of the US 1900 Census (with information on month of birth) was used for comparisons with the general population [44].

### 2.5. Research Design

This study explored the effects of month of birth on the likelihood of survival to age 100. Centenarians (cases) were compared to their “normal” shorter-lived siblings (controls) or spouses using a within-family analysis. This approach allows investigators to study the within-family differences, not being confounded by the between-family variation. Long-lived persons born in 1880–1895 were used as cases. Siblings were born in a wider time window than centenarians but on average in the same year. Taking into account relatively high child mortality in the 19th century, we conducted analyses with different lifespan cut-offs in order to study late-life survival to advanced ages and evaluate the stability of results. The main approach used in this study is based on the comparison of children within rather than across families. A similar approach was applied for comparison of centenarians to their spouses.

### 2.6. Statistical Analyses

Differences in the month-of-birth distributions between centenarians or their siblings and the general population according to the 1900 US Census were assessed with the chi-square test. Standardized residuals were calculated in order to determine which months of birth may be major contributors to rejection of the null hypothesis (in the case it is rejected). When the absolute value of the residual is greater than 2.00, it indicates that it was a major influence on a significant chi-square test statistic. The chi-square test was also used to examine whether gender or longevity is related to the month of birth.

Statistical analyses of the within-family effects for 1 : n matched study were performed using a conditional multiple logistic regression model (fixed-effect model) to investigate the relationship between an outcome of being a case (long-lived person) and a set of prognostic factors [46, 47]. The likelihood of survival to advanced ages (to be in the centenarian group) is used as a dependent variable and month of birth and gender are used as explanatory variables. All analyses were conducted using Stata statistical software, release 11 [48]. Adjustment for multiple comparisons was conducted by the Bonferroni method. However, the Bonferroni adjustment is often criticized by statisticians as being too conservative [49, 50]. A technique proposed by Benjamini and Hochberg offers a more powerful alternative to the traditional Bonferroni method [51]. This technique is based on controlling the false discovery rate (FDR)—the proportion of significant results that are actually false positives. According to the Benjamini and Hochberg procedure, the null hypothesis is rejected when ordered individual *P* values (from smallest to largest) are lower than (*i*/*m*)*Q*, where *i* is a rank of *P* value, *m* is the total number of tests, and *Q* is the chosen FDR. The level of FDR in the Benjamini and Hochberg procedure was set to 0.10.

## 3. Results

**Figure 1:**
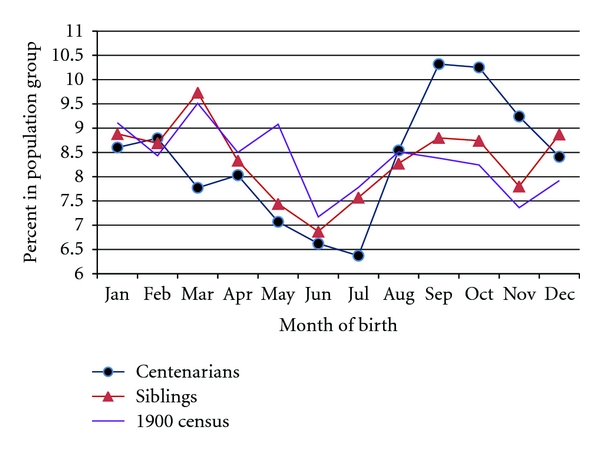
Distribution of individuals by month of birth in percent: centenarians, their shorter-lived siblings survived to age 20 and the USA population born in 1880–1895 according to the 1900 US Census.

Comparison of month-of-birth distributions for centenarians and their shorter-lived siblings with month-of-birth distribution for persons born in 1880–1890 and enumerated by the 1900 US Census showed statistically significant differences (*P* < 0.001 for both centenarians and their siblings).  Table 4 shows month-of-birth distribution for centenarians, their siblings survived to adulthood and the general population. In the case of centenarians, absolute values of standardized residuals exceeded the critical value of 2 in six cases: there is an excess of centenarians born in September–November and a lack of centenarians born in March, May, and July.  Figure 1 shows that the excess of centenarians born in the fall months is particularly high compared to the general population. For siblings, absolute values of standardized residuals exceed the critical value only for May-born and December-born individuals. Overall, the seasonal pattern of births for siblings is closer to that for the general population compared to the seasonal pattern of births for centenarians (Figure 1). In the general population, more persons were born in the first half of the year (51.8%) while more centenarians were born in the second half of the year (53.12%). Centenarian siblings occupy an intermediate position with 49.94% being born in the first half of the year. These differences in birth seasonality (being born in the first or the second half of a year) between centenarians and their shorter-lived siblings (survived to age 20) are statistically significant (chi-square test statistic = 5.03, *df* = 1; *P* = 0.025). As shown in Table 4 and Figure 1, month-of-birth distribution for centenarians also departs from the distribution of their shorter-lived siblings and this difference is statistically significant (all siblings: chi-square test statistic = 19.99, *df* = 11, *P* = 0.045; siblings survived to age 20: chi-square test statistic 19.50, *df* = 11, *P* = 0.053). At the same time, we found no statistically significant association between month of birth and gender.

To analyze the effects of month-of-birth on exceptional longevity, which are not confounded by birth and infant death seasonality, childhood conditions, or genetic background, a within-family study was conducted. To discriminate between the effects due to differential survival early in life from the late-life effects, we analyzed survival to age 100 among siblings conditional on their survival to different adult ages.  Table 5 presents the odds ratios to become a centenarian for siblings born in different months and survived to 30, 50, and 70 years of age. These results demonstrate that persons born in September–November have significantly higher chances of exceptional longevity than persons born in March. This survival advantage of persons born in the fall months is consistent across different lifespan cut-offs suggesting long-lasting influence of season of birth on longevity. 

**Figure 2:**
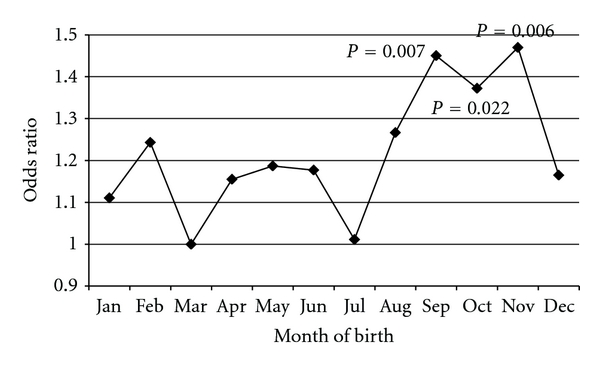
Month of birth and odds ratios for becoming a centenarian. A within-family-study of centenarians and their siblings survived to age 50 (9,724 studied persons). Being born in March is used as a reference level. Unadjusted *P* values are shown.

Being born in the spring months was associated with decreased chances of survival to age 100 while birth in the fall months significantly increases chances to become a centenarian.  Figure 2 depicts the general pattern of month-of-birth effects on longevity after age 50. This pattern suggests that persons born during the fall months have higher chances of survival to age 100 compared to March-born individuals who have the lowest chances of achieving longevity. July-born individuals also show low odds of survival to age 100 compared to individuals born in fall. 

It was suggested that month-of-birth effects on mortality may become weaker for later-born cohorts [19]. To test whether the season-of-birth effects are weaker in later-born cohorts, we split the sample of centenarians and siblings into two approximately equal groups: those who were born before 1899 and those who were born after this year. The effects of month-of-birth on survival after age 50 for these two cohorts are presented in Table 6. For group born before 1899, the odds of survival to 100 are significantly higher for persons born in November compared to persons born in March. For later-born cohorts, the month-of-birth effect is much weaker and not statistically significant after adjustment for multiple comparisons. 

In order to control for living conditions during the adult life, we compared centenarians with their spouses. The results of these comparisons are shown in Table 7 and confirm the month-of-birth pattern in longevity found in previous analyses. Again, individuals born in October-November have a significantly higher likelihood of survival to age 100 compared to individuals born in April. These effects are long-lasting and can be visible after age 50. 

## 4. Discussion

### 4.1. Comparison with Previous Studies

We found that persons born in the fall months are more represented among centenarians compared to the general population while persons born in the first half of the year are less represented among the group of long-lived individuals. Centenarians, their siblings, and the general population show decreased proportion of persons born during the summer months, which is probably related to seasonal distributions of births and infant deaths in the past [52]. The month-of-birth pattern among centenarians in this study is compared to the month-of-birth distribution of persons aged 5–20 years in the US 1900 Census and hence the results of this comparison are not affected by seasonal distribution of infant deaths. In the previous study of German semi-supercentenarians, seasonal distribution of birth dates of long-lived individuals was compared to the seasonal distribution of births 105 years earlier, so this comparison may be influenced by seasonality of infant deaths in the past. At the same time, our results demonstrate some similarity with the results of semi-supercentenarian study: persons born in the second half of the year are over-represented among German semi-supercentenarians [23] as it was shown in this study. The within-family multivariate analysis demonstrated a survival advantage of individuals born in September–November compared to individuals born in March. A similar pattern of season-of-birth and longevity was also found for spouses of centenarians, which reinforces the findings obtained for centenarian siblings. These results are in agreement with previous publications on the effects of month-of-birth on lifespan in the countries of the Northern hemisphere [15, 16, 21, 22, 53] and in the United States in particular [19, 54]. These earlier studies show better survival for persons born in September–December compared to persons born in the middle of the year. 

At the same time, the results of this study show that individuals born in March or April have similar low odds of achieving longevity as individuals born during the summer months and persons born during the winter months do not live longer than the March-born individuals. This is different from the results of other studies, which showed decline in mean age at death for persons born during the summer months and relatively high mean age at death for persons born during the winter months [19, 21, 22]. These differences in month of birth pattern between our study and other publications can be partially explained by changes in seasonality of births and infant deaths over time. Births usually peak in March and hence March-born individuals are overrepresented among both living and dead persons (this is the reason why March-born individuals are highly represented in the general population, see Figure 1). Studies based on the analysis of cross-sectional death certificates do not have information about population at risk [19] and hence may be affected by secular changes in seasonality of births and infant deaths. Although these secular effects probably do not significantly modify the overall month-of-birth pattern in life expectancy, they can affect amplitudes of seasonal effects for specific months. It would be reasonable to suggest that decline of summer infant deaths over time resulted in increased representation of summer-born individuals in the later-born cohorts, which led to an apparent drop in the mean age at death for persons born in these months. 

Study of the earlier-born and the later-born groups found that the season-of-birth effects fade in the later born cohorts (Table 6), which is consistent with previous reports [19] and can be explained by improving nutrition and sanitation over time. We found no gender differences in month-of-birth distributions for both centenarians and their siblings, which is consistent with previous publications [19]. 

It should be noted that another study of season-of-birth effects on life span in the single-year USA birth cohorts (based on the USA Social Security Administration data) found that life expectancy at age 80 depends on month of birth [54]. In this study, 80-year olds born in May-June showed significantly lower life expectancy compared to individuals born in the end of the year and this seasonal pattern repeats itself in every studied birth cohort. This month-of-birth pattern of life expectancy is similar to the pattern reported earlier for mean age at death obtained on the basis of the USA death certificates [19]. However, in the study of centenarians and their siblings, we do not find a specific survival advantage for persons born in the winter months. It is possible that certain unobserved socioeconomic or other characteristics of parents (such as possible preferential winter births for wealthier social groups), which are controlled for in the case-sibling design of our study, may result in apparently better survival of winter-born individuals in the general population. Further research is needed for better explanation of this phenomenon. 

### 4.2. Strengths and Limitations

Our within-family study follows centenarians and their siblings from birth until the end of their life while previous studies analyzed a cross-sectional sample of the USA death certificates for persons belonging to multiple birth cohorts. For this reason, our results do not depend on the secular changes in seasonality of births and infant deaths. Another advantage of this study is its within-family design, which controls for unobserved characteristics of childhood conditions and parental genetic background. This study confirms the existence of month-of-birth effects on longevity and shows that these effects can be observed even after controlling for unobserved between-family variation. 

Some limitations of this study should be mentioned. Due to the data collection from computerized genealogies, we cannot be certain that centenarians (and controls) represent a random population sample. This limitation is not crucial for the analytical approach applied in this study, which tests specific hypothesis of seasonal birth effects on longevity, but may pose a question about generalizability of results. It is believed that the RootsWeb source of online family histories has more individuals with larger families and better offspring survival. Indeed our sample of centenarians has larger families compared to the general population. This deviation from the general population may potentially affect the results of univariate analyses when month-of-birth distributions for centenarians and siblings are compared to the general population. However, in the within-family analyses, we compare siblings with each other rather than with the general population, so the difference in family size does not affect the results of hypothesis testing about the month-of-birth effects on longevity [55]. Also, comparison with the general population shows better similarity of month-of-birth pattern for siblings rather than centenarians suggesting that shorter-lived siblings are closer to the general population in terms of month-of-birth distribution. 

Another problem is that some month-of-birth effects become not statistically significant after adjustment for multiple comparisons. For example, month-of-birth effects become nonsignificant when survival of siblings after age 70 is studied. However, the overall pattern of month-of-birth effects on longevity shows consistency across different age cut-offs suggesting a stability of the observed seasonal pattern. In addition to that, independent analyses on centenarian spouses demonstrated a similar pattern of month-of-birth effects on longevity. Finally, the conditional logistic regression analyses suggest that despite significant effects of months of birth on relative survival the effect sizes of month-of-birth effects on survival to 100 are small and explain about 2% of the variance of becoming a centenarian. This small percentage of explained variance is related to very high variability of individual lifespan, which has a substantial stochastic component [6]. 

### 4.3. Existing Explanations of Month-of-Birth Effects on Longevity

#### 4.3.1. Maternal and Child Nutrition in the Past

There are several possible explanations of why month of birth may affect mortality and health later in life. One explanation suggests that nutritional status of mother during pregnancy may affect fetal development in utero [3, 56]. Nutritional deficiencies during early development may have long-lasting effects on mortality later in life [3]. This explanation is supported by the Ames theory [57] that micronutrient deficiencies play a major role in DNA damaging, human aging, and premature deaths from cancer and heart disease. Recent review suggests that both improper diet stimulating chronic inflammation and dietary deficiencies and nutrient imbalances may be strong sources of mutagenesis [58]. So it is reasonable to hypothesize that seasonal vitamin deficiency during the critical periods of fetus and infant development may affect later health and longevity of the deficiency-exposed birth cohorts. 

Birth weight often serves as an indicator of nutritional status during early development and was shown to be dependent on month of birth. For example, in Greece, infants born during the autumn and winter seasons of the year had significantly increased birth weight and gestation age [20]. Recent review of birth weight seasonality in developed countries shows a tendency of infants born during the fall and winter seasons in European countries to have higher birth weights [59]. There are also reports that premature births show a slight excess of incidence during the months of June–August [60]. 

#### 4.3.2. Seasonal Infections

Early seasonal impacts on subsequent adult lifespan may include not only seasonal vitamin deficiency, but also other seasonal impacts, such as infectious diseases. Seasonal peaks of disease occurrence are typical for many infections [61]. The most drastic effects of infectious agents in pregnancy, which probably represent the tip of the iceberg of the damage to progeny, include the following: cardiac malformation, deafness, cataracts, glaucoma, and tooth defects for the rubella virus (German measles); growth retardation, blindness, mental retardation, and deafness for cytomegalovirus; skin scarring, muscle atrophy, and mental retardation for varicella (chickenpox) [62, 63]. It was shown that poliovirus epidemics peak in July-August and exposure to this virus in the second trimester of gestation seem to produce subsequent adult schizophrenia in February birth cohorts [64]. 

#### 4.3.3. Environmental Temperature at Birth and Conception

Effect of environmental temperature during the time of birth or conception may be another possible explanation for low proportion of centenarians among individuals born during the summer and spring months. For example, British women experiencing higher summer temperatures during their first year of life and hence suffering severe diarrhea and dehydration in infancy had higher blood pressure at older ages [65]. High ambient temperature was also associated with higher risk of preterm delivery in the recent study of a large sample of California births [66]. There are reports that high temperatures may be implicated in lower sperm quality [67, 68], particularly among smokers [69]. This may result in a less viable progeny born during the spring months. On the other hand, cold outdoor temperature at birth during the winter months is associated with coronary heart disease, insulin resistance, and poor lung function at older age [70]. 

#### 4.3.4. The Deadline Hypothesis

It was suggested that schools or other professional training organizations, which have a deadline for admission, may favor children who are somewhat older compared to their peers (usually children born in the fall months). This so-called deadline hypothesis [19] predicts that the relative advantage in school achievement has cumulative effects over the life course. In the case of historical data, the deadline hypothesis should be more relevant to survival of men whose social status is dependent on their own achievements. For women, their social status in the past was predominantly determined by the social status of their husbands. In the case of centenarians, who are predominantly women, the deadline hypothesis looks like a less likely explanation of the observed month-of-birth effects on longevity. 

### 4.4. Explanation of Survival Advantage for Persons Born in the Fall Months

Analysis of the existing literature suggests that persons born in the fall months in the United States could avoid extremes of very high and very low ambient temperatures during their first month of life as well as high summer temperatures during conception. Persons born during the fall months also did not experience an early exposure to infectious diseases, which were common during summer, early winter, or spring months in the past. Seasonal pattern of the USA mortality for children below age one month in the past supports this suggestion. According to the USA statistics, mortality below age one month in 1940 was the lowest in September–November [52] suggesting lower infectious load during this period of the year, because most infant deaths in the past were caused by infections. Better maternal nutrition during the last trimester of pregnancy also contributed to the survival advantage of individuals born during the fall season. All these three factors (mild ambient temperatures, better maternal nutrition, and low infectious load) helped persons born in the fall months to avoid accumulation of excessive number of defects by body systems very early in life. These results are consistent with the high initial damage load (HIDL) hypothesis [6, 7], which emphasizes the importance of the initial level of damage in determining future human longevity. More specific explanation of the observed month-of-birth effects on longevity can be provided by the inflammation hypothesis suggested by Finch and Crimmins [11]. According to this hypothesis, a strong acute-phase inflammatory response to childhood infections initiates chronic inflammation, which promotes chronic diseases of aging. Reduced lifetime exposure to infection and subsequent inflammation may explain both declining mortality at older ages and decreasing amplitude of month-of-birth effects on lifespan over time. The results obtained in this study suggest that optimizing the process of early development can potentially result in avoiding many diseases in later life and significant extension of healthy life span. More research is needed to determine more specific factors of seasonal birth effects on longevity.

## 5. Conclusions

This is the first study of association between month of birth and exceptional longevity, which controls for early-life shared conditions and common genetic background. We developed a large sample of validated centenarians, their siblings, and spouses to study early-life seasonal effects on human longevity. We found significant associations between month of birth and longevity: individuals born in September–November have higher likelihood of becoming centenarians compared to March-born individuals. These results are consistent with the reports of higher life expectancy for persons born in the end of the year [16, 19, 21, 22] and the study of mortality after age 80 in several single-year USA birth cohorts [54]. The results of this study demonstrate that month-of-birth effects on exceptional longevity persist after controlling for shared childhood environment and unobserved shared characteristics of parents. Association of month-of-birth with exceptional longevity appears to be stronger for earlier birth cohorts born before 1899. Similar month-of-birth effects on longevity were found for centenarian spouses: individuals born in October-November were more likely to live to 100 compared to individuals born in April. The results of this study suggest that early-life environmental conditions may have long-lasting effects on human aging and longevity.

## Figures and Tables

**Table 1:** Number of centenarians and their siblings at different stages of data collection and cleaning.

Type of records	Centenarians			Number of shorter-lived siblings
	Males	Females	Total	
All initial nonduplicate records for centenarians born in 1880–1895 with names of parents available	7,174	18,277	25,451	
Centenarians having detailed information on birth and death dates of their parents	6,370	16,757	23,127	172,091
Centenarians having detailed information on birth and death dates of their parents and siblings	707	2,127	2,834	21,893
Centenarians after data cleaning with confirmed death dates through the linkage to DMF	365	1,209	1,574	10,885

**Table 2:** Distribution of centenarians born in 1880–1895, by age at death.

Age at death	Centenarians having siblings		
	Men	Women	Both sexes
100	132	398	530
101	92	266	358
102	52	214	266
103	43	137	180
104	16	71	87
105	18	58	76
106	9	38	47
107	2	15	17
108	0	5	5
109	1	3	4
110	0	3	3
111	0	0	0
112	0	1	1

Total:	365	1,209	1,574

**Table 3:** Characteristics of centenarians born in 1880–1895 and their siblings and spouses. Values are numbers (percentages) or means (standard deviations).

Characteristic	Men	Women	Both Sexes
Number of records (percent)			
Centenarians, total	365 (23.2)	1,209 (76.8)	1,574 (100.0)
Centenarians with spouses	231 (23.9)	737 (76.1)	968 (100.0)
Siblings of centenarians	5,731 (52.7)	5,154 (47.3)	10,885 (100.0)
Spouses of centenarians	814 (75.2)	269 (24.8)	1,083 (100.0)

Mean age at death, years (standard deviation)			
Centenarians, total	101.5 (1.7)	101.8 (1.9)	101.7 (1.9)
Centenarians with spouses	101.5 (1.8)	101.8 (2.0)	101.8 (1.9)
Siblings of centenarians	62.9 (29.3)	66.1 (30.7)	64.3 (29.9)
Spouses of centenarians	72.69 (14.7)	77.8 (17.1)	73.9 (15.5)

Mean year of birth (standard deviation)			
Centenarians, total	1887.0 (5.5)	1888.6 (5.5)	1888.2 (5.5)
Centenarians with spouses	1887.4 (5.0)	1888.8 (4.7)	1888.4 (4.8)
Siblings of centenarians	1888.6 (10.4)	1889.0 (10.3)	1888.8 (10.4)
Spouses of centenarians	1885.4 (7.5)	1892.2 (7.2)	1887.1 (8.0)

**Table 4:** Month-of-birth distributions (in percent) for the US 1900 Census population, centenarians, and their siblings^a^.

Month of birth	1900 Census, 5% sample Persons born in 1880–95	Centenarians		Siblings survived to age 20	
	*N* = 1,320,328	*N* = 1,570	Standardized residuals	*N* = 9,175	Standardized residuals
January	9.11	8.60	−0.671	8.88	−0.721
February	8.43	8.79	0.491	8.69	0.847
March	9.51	7.77	−2.235	9.73	0.693
April	8.50	8.03	−0.645	8.33	−0.568
May	9.08	7.07	−2.643	7.44	−5.200
June	7.17	6.62	−0.808	6.87	−1.086
July	7.78	6.37	−2.004	7.57	−0.704
August	8.51	8.54	0.034	8.27	−0.780
September	8.38	10.32	2.653	8.80	1.375
October	8.24	10.25	2.781	8.74	1.672
November	7.36	9.24	2.739	7.80	1.567
December	7.92	8.41	0.687	8.87	3.240

^a^Month-of-birth distributions for both centenarians and their siblings differ from the month-of-birth distribution for the general population (individuals enumerated in the 1900 census and born in 1880–1895); difference significant at *P* < 0.001.

**Table 5:** Odds ratios (*P* values) to become a centenarian as predicted by conditional logistic regression (fixed effects) for different age cut-off subgroups. Effects of month of birth^a^.

Variable	All siblings	Siblings survived to age 30	Siblings survived to age 50	Siblings survived to age 70
Month of birth:				
January	1.13 (0.387)	1.11 (0.472)	1.11 (0.463)	1.09 (0.537)
February	1.25 (0.101)	1.25 (0.109)	1.24 (0.124)	1.16 (0.303)
March	Reference	Reference	Reference	Reference
April	1.15 (0.320)	1.15 (0.337)	1.16 (0.320)	1.09 (0.567)
May	1.20 (0.218)	1.17 (0.288)	1.19 (0.251)	1.15 (0.373)
June	1.20 (0.229)	1.00 (0.254)	1.18 (0.284)	1.11 (0.486)
July	1.03 (0.855)	1.19 (0.991)	1.01 (0.941)	1.00 (0.990)
August	1.25 (0.110)	1.24 (0.125)	1.27 (0.100)	1.21 (0.198)
September	**1.44 (0.006)** ^ c^	**1.43 (0.009**)^c^	**1.45 (0.007)** ^ c^	**1.39 (0.022)**
October	**1.43 (0.008)** ^ c^	**1.37 (0.021)** ^ c^	**1.37 (0.022)** ^ c^	1.27 (0.099)
November	**1.51 (0.003)** ^ b,c^	**1.48 (0.005)** ^ c^	**1.47 (0.006)** ^ c^	**1.41 (0.017)**
December	1.17 (0.266)	1.13 (0.380)	1.17 (0.283)	1.11 (0.486)

Female sex	3.77 (<0.001)	3.82 (<0.001)	3.80 (<0.001)	3.41 (<0.001)

Pseudo *R* ^2^	0.0811	0.0861	0.0871	0.0766
Number of observations	12,132	10,393	9,724	8,123

^a^Statistically significant effects (*P* < 0.05) are highlighted in bold.

^b^Statistically significant after Bonferroni adjustment.

^c^Statistically significant after Benjamini-Hochberg procedure.

**Table 6:** Odds ratios (*P* values) to become a centenarian as predicted by conditional logistic regression (fixed effects), by different birth cohort subgroups for siblings survived to age 50. Effects of month of birth^a^.

Variable	Born before 1889	Born in 1889 or later
Month of birth:		
January	1.42 (0.109)	0.90 (0.628)
February	1.41 (0.118)	1.02 (0.911)
March	Reference	Reference
April	1.09 (0.717)	1.18 (0.467)
May	1.27 (0.312)	0.86 (0.500)
June	1.30 (0.289)	1.35 (0.192)
July	1.14 (0.579)	1.00 (0.984)
August	1.19 (0.446)	1.23 (0.360)
September	1.31 (0.209)	**1.55 (0.042)**
October	**1.61 (0.027)**	1.13 (0.578)
November	**1.78 (0.008)** ^ b^	1.23 (0.357)
December	1.33 (0.185)	0.94 (0.772)

Female sex	3.32 (<0.001)	4.74 (<0.001)

Pseudo *R* ^2^	0.0842	0.1249
Number of observations	3,279	3,441

^a^Statistically significant effects (*P* < 0.05) are highlighted in bold.

^b^Statistically significant after Benjamini-Hochberg procedure.

**Table 7:** Odds ratios (*P* values) to become a centenarian as predicted by conditional logistic regression (fixed effects), by different age cut-off and gender subgroups for spouses of centenarians. Effects of month of birth^a^.

Variable	All spouses	Spouses survived to age 50
Month of birth:		
January	1.27 (0.358)	1.29 (0.346)
February	1.65 (0.066)	**1.83 (0.032)**
March	1.58 (0.084)	1.65 (0.067)
April	Reference	Reference
May	1.37 (0.263)	1.46 (0.190)
June	1.46 (0.176)	1.63 (0.099)
July	1.61 (0.096)	1.66 (0.086)
August	1.52 (0.117)	1.56 (0.112)
September	1.58 (0.079)	1.66 (0.063)
October	**2.17 (0.004)** ^ b,c^	**2.24 (0.004)** ^ b,c^
November	**2.22 (0.003)** ^ b,c^	**2.22 (0.004)** ^ b,c^
December	1.21 (0.487)	1.32 (0.332)

Female sex	3.40 (<0.001)	3.42 (<0.001)

Pseudo *R* ^2^	0.2192	0.2226
Number of observations	1,921	1,800

^a^Statistically significant seasonal effects (*P* < 0.05) are highlighted in bold.

^b^Statistically significant after Bonferroni adjustment.

^c^Statistically significant after Benjamini-Hochberg procedure.
